# Cytology material is equivalent to tumor tissue in determining mutations of *BRCA 1/2* genes in patients with tubo-ovarian high grade serous carcinoma

**DOI:** 10.1186/s12885-019-5535-2

**Published:** 2019-04-02

**Authors:** Andreja Gornjec, Srdjan Novakovic, Vida Stegel, Marko Hocevar, Ziva Pohar Marinsek, Barbara Gazic, Mateja Krajc, Erik Skof

**Affiliations:** 1 Department of Gynecologic Oncology, Institute of Oncology Ljubljana, Zaloška cesta 2, 1000 Ljubljana, Slovenia; 2 Department of Molecular diagnostics, Institute of Oncology Ljubljana, Zaloška cesta 2, 1000 Ljubljana, Slovenia; 3 Department of Surgical Oncology, Institute of Oncology Ljubljana, Zaloška cesta 2, 1000 Ljubljana, Slovenia; 4 Department of Cythopathology, Institute of Oncology Ljubljana, Zaloška cesta 2, 1000 Ljubljana, Slovenia; 5 Department of Pathology, Institute of Oncology Ljubljana, Zaloška cesta 2, 1000 Ljubljana, Slovenia; 6 Department of Genetic counselling, Institute of Oncology Ljubljana, Zaloška cesta 2, 1000 Ljubljana, Slovenia; 7 Department of Medical oncology, Institute of Oncology Ljubljana, Zaloška cesta 2, 1000 Ljubljana, Slovenia

**Keywords:** High-grade serous cancer, *BRCA1/2* mutation, *BRCA1/2* mutation testing, Cytological samples, Formalin fixed paraffin embedded samples

## Abstract

**Background:**

High-grade serous ovarian cancer is a detrimental disease. Treatment options in patients with a recurrent disease are dependent on *BRCA1/2* mutation status since only patients with known BRCA mutation are eligible for treatment with poly(ADP-ribose) polymerase inhibitors (PARPi). The aim of this study was to compare concordance of *BRCA* mutation analyses from cytological samples (CS) with *BRCA* mutation analyses from histological formalin fixed paraffin embedded (FFPE) samples.

**Methods:**

Mutation analysis of *BRCA1* and *BRCA2* genes was performed in 44 women diagnosed with primary or recurrent high-grade ovarian cancer from three different samples: blood, cytological sample (ascites, pleural effusion and enlarged lymph nodes) and tumor tissue. Results from all three samples were compared.

**Results:**

Among 44 patients, there were 15 germline mutations and two somatic mutations. A 100% concordance was found between cytological and histologic samples.

**Conclusion:**

There is a 100% concordance in *BRCA* mutation testing between cytological and histologic samples. *BRCA* mutation testing from CS could replace testing from FFPE tissue in clinical decision making in ovarian cancer patients.

**Trial registration:**

The study was retrospectively registered at ISRCTN registry on 24/11/2015 - ISRCTN42408038.

## Background

Ovarian cancer (OC) is in 3,5–18% associated with germline mutations in *BRCA1* and *BRCA2* genes [1–4]. Germline BRCA1 and BRCA2 mutations are most common in high grade serous histologic type (high grade serous cancer – HGSC), the most frequent type of epithelial ovarian cancer, and appear in 16–39.2% of cases [5–10]. In addition, somatic *BRCA1/2* mutations are present in 3.0–7.9% of cases in ovarian cancer patients [8, 11, 12]. Germline *BRCA* mutations have a positive impact on overall survival and platinum responsiveness of ovarian cancer patients [13]. Somatic BRCA mutations have the same impact [9, 14, 15]. Furthermore, another important hallmark of BRCA1/2-associated ovarian cancers is sensitivity to poly(ADP-ribose) polymerase inhibitors (PARPi) [9].

In Europe, an oral potent PARP inhibitor Olaparib has been added to treatment guidelines for patients with platinum sensitive recurrent serous ovarian, tubal and primary peritoneal cancers with known *BRCA* mutation. Irrespective of whether the origin of the *BRCA* mutation is germline or somatic, PARP inhibitor Olaparib significantly prolongs progression-free survival (PFS) versus placebo in patients with platinum-sensitive recurrent serous ovarian cancer [9, 12, 14]. Before the enrolment of treatment with PARP inhibitor *BRCA* mutation has to be proven (germline or somatic). *BRCA* status is not known in majority of the ovarian cancer patients. Therefor genetic testing must precede enrolment of the treatment.

Germline *BRCA* mutation testing is at this time done only after the genetic counseling in patients with positive familial history for inherited breast and ovarian cancer syndrome and in some patients with known breast or ovarian cancer. Aim of this genetic testing is primary and secondary preventive. Germline *BRCA* mutations are analyzed from peripheral blood cells. Somatic *BRCA* mutation tests have to be done from tumor cells.

Due to the intratumoral genetic heterogeneity (ITH) in primary and recurrent cancers, [16–18] intratumoral widespread regional diversities observed in mutational, copy number and gene expression landscapes using derived samples of primary tumor and metastatic sites [19] and importance of presence of somatic mutations, [15, 18, 20–23] the best strategy for *BRCA* mutation testing would be from tumor cells. Tumor cells for *BRCA* testing can be obtained from histologic samples gained during operative procedures or from cytological material derived from minimally invasive procedures. Genetic testing from histologic samples is usually performed from formalin-fixed paraffin-embedded (FFPE) tissue samples, where quality of isolated DNA could be a problem. Genetic testing would be ideally done from fresh or fresh frozen tissue samples. In most of the cases it is not feasible, because of spatial and logistic problems and time divergence of genetic testing and gaining of tissue samples, which are most of the times done after primary or interval cytoreduction [24–26]. On the other hand genetic material extracted from cytological samples is of good quality and relatively easy accessible, which enables genetic testing. Ovarian cancer is in 75% of patients diagnosed at advanced stages where tumor has already spread to peritoneal surfaces and cells are present in peritoneal free fluid. Recurrent disease often presents with malignant ascites and peritoneal and abdominal metastases and is seldom treated with operative procedure where tissue samples could be taken. Cytological material is easily accessible before nearly every treatment enrolment without invasive procedures such as surgical operation. To our knowledge, only two studies did genetic analysis on DNA isolated from ascites derived cancer cells and one on DNA isolated from cells in pleural effusions of patients with HGSC. Castellarin et all performed whole exome sequencing on tumor cells harvested from ascites. They provided the first evidence at single nucleotide resolution that recurrent high grade serous cancer arises from multiple clones present in the primary tumor with negligible accumulation of new mutations during standard treatment [16]. Results from Choi et all confirmed the theory that ascites cells are shed from solid tumor lesions and showed that ascites derived cancer cells integrated ITH and found that mutation profiling results of ascites cells in the ovarian HGSC patients represent and integrate entire mutational landscape of a given HGSC and cover the majority of somatic mutations found across regional biopsies of the corresponding tumors [22]. The study from Shah et all compared genomic alterations results from DNA extracted from pleural effusion, FFPE and matched blood from 4 HGSC patients. They confirmed that cytological material could represent good material for genomic alterations identification with next generation sequencing and demonstrated that very little DNA (<50 ng) can yield extremely high coverage sequence data [18]. Considering all characteristics of HGSC as disease with lots of recurrences, eventual chemoresistance, somatic reverting mutations and also ITH with limited availability of FFPE before every treatment enrolment, knowing that targeted personalized therapy is future directive for HGSC treatment and with confidence data that ascites tumor cells represent entire mutational profiling landscape of given HGSC, cytology could be equivalent to FFPE and even represent the most accurate mutation profiling result for a given HGSC in a certain time of disease progression.

The aim of our study was to compare *BRCA* mutation profiling of cytological material (cells in ascites, cells from pleural effusion or cells derived from fine needle aspiration of lymph node metastasis) with histologic samples from tumor tissue obtained during operation.

We expected to confirm 100% concordance between *BRCA* mutation testing from cytological samples (CS) in comparison to FFPE. We wanted to determine if *BRCA1/2* testing from CS can be used instead of FFPE in HGSC patients.

## Methods

From October 2015 to December 2017 patients with primary or recurrent ovarian, fallopian tube and primary peritoneal high-grade serous cancer (subsequently collectively referred to as high-grade serous cancer – HGSC) who were treated at the Institute of Oncology Ljubljana, were included in the study.

The study was approved by National Ethics Committee and Institutional Ethics Committee of Institute of Oncology Ljubljana (Ljubljana, Slovenia), 27/07/2015, NMEC 100/05/15. All samples from the patients in this study were obtained after appropriate informed written consents. The study was retrospectively registered at ISRCTN registry on 24/11/2015 (ISRCTN42408038).

Inclusion criteria were as follows: age < 70 Years, WHO performance status 0–1, histologically verified high grade serous ovarian, tubal or primary peritoneal cancer, presence of ascites or enlarged peripheral lymph nodes, availability of FFPE tissue samples derived at surgical operation, indication for enrolment of treatment with platinum agents, normal kidney function (creatinine <100 μmol/l, endogenous creatinine clearance (ECC) > 75 ml/l).

Exclusion criteria were as follows: types other than HGSC of ovarian cancer, unavailability of FFPE tissue samples derived at surgical operation, no malignant cells in ascites or enlarged peripheral lymph nodes, WHO performance status≥2, history of presence of other cancer (except squamous cell skin cancer, uterine cervical cancer, breast cancer and in situ breast cancer).

Only patients with available three sample types (CS, blood and FFPE) were considered eligible.

All included patients performed genetic counselling and testing for germline BRCA1/2 mutation at Institute of Oncology Ljubljana.

### Cytological samples evaluation

Cytological samples were obtained either at ultrasonography guided transabdominal puncture, with thoracic paracentesis or with fine needle biopsy in ambulatory setting under local anesthesia. Samples (at least 30 ml of ascites or pleural effusion) were promptly sent to the Cytology department. All included patients (including patients with primary inoperable disease who were treated with neoadjuvant chemotherapy) had confirmed disease (HGSC) by FFPE – described in inclusion/exclusion criteria. Ascites and pleural effusion cells were pelleted by classical centrifugation (2700/10 min). Two smears were prepared from each sample according to our standard protocol and stained with Giemsa or Papanicolau. To a part of each sample a cell medium (20% bovine serum albumin, 5% EDTA, 4 N NaOH in phosphate buffer solution with 1000.000 IE of penicillin and 80 mg Garamycin), routinely prepared in our laboratory, was added. Cell medium was also added to fine needle punctate of enlarged lymph nodes. Sample was cytocentrifugated and prepared for cythopathological review. One cythopathologist reviewed all prepared samples and assessed the presence of adenocarcinoma tumor cells in the sample. Samples were then sent to Molecular department for *BRCA* testing.

### Histological samples evaluation

Histological samples were obtained at interval cytoreductive surgery or at primary surgical operation and sent to the Pathology department. All samples were formalin fixed and paraffin embedded. Hematoxylin and Eosin (HE) stained slides were prepared from each paraffin block. One pathologist reviewed all HE-slides of each patient, confirmed the tumor type, evaluated the percentage of tumor cells on each slide and chose a representative paraffin block where at least 60% of tumor tissue was present. From the selected block samples were prepared for molecular analysis and sent to the Department of Molecular Diagnostics for *BRCA* testing.

### Extraction of DNA

In the study the DNA samples extracted from whole blood, ascites and formalin-fixed paraffin embedded tumor tissue were used.

From the whole blood and ascites, the DNA was extracted using InnuPREP Master Blood kit (Analytik Jena, Thuringia, D). From FFPE tumor tissue, the DNA was extracted using GeneRead DNA FFPE Kit (QiagenGmbH, Hilden, Germany)) from manually macro-dissected areas annotated by a pathologist by scraping directly off unstained standard glass slides (10 μm). Hematoxylin-eosin staining of the first sectioned slide was performed to visualize the presence of tumor cells, and to guide macro-dissection on unstained duplicate slides and to determine the area of the tissue cores. After the isolation DNA concentration was spectrophotometrically measured at 280/260 nm.

### Next generation sequencing - NGS

The coding sequence and exon/intron boundaries on DNA isolated from blood and ascites were enriched using Nextera DNA Library Preparation Kit in combination with TruSight® Cancer Panel (Illumina, San Diego, USA). The coding sequence and exon/intron boundaries of *BRCA1* and *BRCA2* genes on DNA isolated from FFPE tumor tissue and ascites were amplified using TruSeq Custom Amplicon Kit in combination with primer pools in Targeted DNA Repair_2 Gene consortium panel kit *–* TSCA-BRCA (Illumina, San Diego, USA). Before the library preparation the DNA quality and quantity was assessed using Infinium FFPE QC and DNA Restoration Kit (Illumina). Next generation sequencing was performed on Illumina MiSeqDx Sequencing System (Illumina) according to manufacturer’s protocol. Secondary data analysis and base calling was performed by MiSeq Reporter Software 2.5.1, using DS Somatic as variant caller (Illumina). VCF v4.1 files generated during secondary analysis of sequencing data were imported into Illumina Variant Studio software for variant annotation and filtering. When TruSight® Cancer Panel was used, more than 95% of targeted regions in *BRCA1* and *BRCA2* genes were covered >150x. Estimated limit of detection for this method was 10% of mutant allele in the background of wild type for single nucleotide variants (SNV), and 25% for insertion and deletions (indel) (up to 11 bp). When TSCA-BRCA was used, more than 95% of targeted regions in *BRCA1* and *BRCA2* genes were covered >500x. Estimated limit of detection for this method was 5% for SNV and 10% for indel (up to 11 bp).

Classification of detected variants was done through Illumina Variant Interpreter Software (Illumina).

In silico mutation prediction analysis was performed applying online bioinformatics tool Alamut® Visual 2.11.

### Direct sequencing

Direct Sanger sequencing was performed to validate mutations detected by NGS. For direct DNA sequencing, the samples were bidirectionally sequenced on an automated ABI 3500 genetic analyzer (Applied Biosystems, Foster City, CA). Estimated limit of detection is 20% of mutant allele in the background of wild type for SNV and indels (up to 11 bp).

### MLPA analysis

For detection of large deletions and insertions the MRC Holland MLPA (Multiplex Ligation – dependent Probe Amplification) kit P045-B1 for *BRCA2*, and P002 for *BRCA1* was used according to manufacturer’s instructions (MRC Holland, Amsterdam, Nederland).

Nucleotide variants were classified according to ACMG guidelines [27, 28].

### Statistical analysis

The study was designed to have 80% power of research, to prove ≥90% certainty of cytology, knowing that cytology specificity is at least 99%, where risk for α error is 5% or less. To meet these criteria, we had to include at least 40 patients [29].

Descriptive statistics was used to describe basic features of the data. For differences of BRCA mutation test results between CS, FFPE and blood group Chi^2^ test was used. *P* value < 0,05 was considered significant.

Statistical analysis was done with IBM SPSS®25 software.

## Results

Overall 44 patients with HGSC and results of *BRCA* testing from three sample types (CS, blood and FFPE) were included in this study. Their clinical characteristics are presented in Table 1. Mean age at the time of diagnosis was 59.8 years (range 40–77). Eight out of 44 patients (18.1%) had a positive family history for hereditary breast and ovarian cancer and 6/44 (13.6%) had a known diagnosis of breast cancer before HGSC was diagnosed. Four out of these six patients (66.7%) had also a positive familial history. Mean time from breast cancer diagnosis to ovarian cancer diagnosis was 21,33 years (range 9–28). There was not a single breast cancer relapse in these patients and so free interval from breast cancer to ovarian cancer was more than 20 years. All clinical characteristics of disease including serum tumor markers at the time of sample collection were in accordance with ovarian cancer.

**Table 1:** Clinical characteristics of 44 patients with HGSC

Age at diagnosis	years
Mean	59,8
Range	40–77
Localisation of HGSC at diagnosis	n (%)
Ovarian cancer	34 (77,3)
Fallopian tube cancer	6 (13,6)
Primary peritoneal serous cancer	4 (9,1)
FIGO Stage	n (%)
IA	0 (0)
IB	1 (2,3)
IC	2 (4,5)
II	0 (0)
IIIA1	1 (2,3)
IIIC	30 (68,2)
IV	10 (22,7)
Family history	n (%)
Positive	8 (18,2)
Negative	34 (77,3)
Unknown	2 (4,5)
Breast cancer	n (%)
Yes	6 (13,6)
No	26 (59,1)
Unknown	12 (27,3)

Legend: *HGSC* high grade serous cancer, *FIGO* International Federation of Gynecology and Obstetrics

### *BRCA* mutation testing results

#### Blood *BRCA1/2* testing results

Fifteen *BRCA1/2* mutations (15/44, 34.1%) were identified in peripheral venous blood. Fourteen patients had a *BRCA1* mutation and one had a *BRCA2* mutation. All other patients had wild type *BRCA* genes. All mutations were known pathogenic mutations. The most common *BRCA1* mutation was c.843_846delCTCA p.(Ser282Tyrfs*15) which was present in three patients. *BRCA1* mutations c.844_850dupTCATTAC p.(Gln284Leufs*5), deletion of exons 4–9 and c.1687C > T p.(Gln563*) were all present in two patients. All other mutations were present in only one patient (Table 2).

**Table 2:** *BRCA* mutations detected in blood, FFPE tumor samples and cytological samples of ovarian cancer patients

Sample No.	*Gene*	Mutation	Mutation detected in blood by TSCancer^a^	Mutation detected in FFPE tumor sample by TSCA^a^	Mutation detected in cytological sample by TSCancer^a^	Mutation detected in cytological sample by TSCA^a^	CS sample type^b^	Germline/somatic mutation
1	*BRCA1*	c.844_850dupTCATTAC p.(Gln284Leufs*5)	YES	YES	YES	YES	AS	germline
2	*BRCA1*	c.116G > A p.(Cys39Tyr)	YES	YES	YES	YES	AS	germline
3	*BRCA1*	del ex 4–9 c.(134 + 1_135–1)_(670 + 1_671–1)del p.	YES	YES	YES	YES	AS	germline
4	*BRCA1*	c.1543G > T p.(Glu515*)	NO	YES	YES	YES	ELN	somatic
5	*BRCA1*	c.3018_3021delTTCA p.(His1006Glnfs*17)	YES	YES	YES	YES	PE	germline
6	*BRCA1*	c.1687C > T p.(Gln563*)	YES	YES	YES	YES	AS	germline
7	*BRCA1*	c.850C > T p.(Gln284*)	YES	YES	YES	YES	AS	germline
8	*BRCA1*	del ex4–9 c.(134 + 1_135–1)_(670 + 1_671–1)del p.?	YES	YES	YES	YES	ELN	germline
9	*BRCA2*	c.4139_4140dupTT p.(Lys1381Leufs*8)	YES	YES	YES	YES	AS	germline
10	*BRCA1*	c.843_846delCTCA p.(Ser282Tyrfs*15)	YES	YES	YES	YES	ELN	germline
11	*BRCA1*	c.843_846delCTCA p.(Ser282Tyrfs*15)	YES	YES	YES	YES	ELN	germline
12	*BRCA2*	del ex22–27 c.(8754 + 1_8755–1)_(*1_?)del p.?	NO	YES	YES	YES	ELN	somatic
13	*BRCA1*	c.843_846delCTCA p.(Ser282Tyrfs*15)	YES	YES	YES	YES	PE	germline
14	*BRCA1*	c.1687C > T p.(Gln563*)	YES	YES	YES	YES	AS	germline
15	*BRCA1*	c.3436_3439delTGTT p.(Cys1146Leufs*8)	YES	YES	YES	YES	AS	germline
16	*BRCA1*	c.844_850dupTCATTAC p.(Gln284Leufs*5)	YES	YES	YES	YES	AS	germline
17	*BRCA1*	c.4065_4068delTCAA p.(Asn1355Lysfs*10)	YES	YES	YES	YES	AS	germline

*CS* cytological sample, ^a^Method used for NGS sequencing; ^b^*AS* ascitic fluid, *ELN* enlarged lymph node, *PE* pleural effusion, *TSCancer* TrueSight Cancer panel, *TSCA* TruSeq Custom Amplicon Kit

#### Cytological *BRCA1/2* testing results

Cytological samples for DNA analysis were derived from ascites fluid (AS) in 34, enlarged lymph nodes (ELN) in 6 and pleural effusion (PE) in four patients (Table 1). In 20 patients cytological samples were taken before primary treatment of newly diagnosed disease and in 24 patients before enrolment of treatment of recurrent disease. Percentage of tumor cells present per sample was on average 53.1% (range 1–95%). Seventeen *BRCA1/2* mutations were identified in cytological samples: 15 *BRCA1* and 2 *BRCA2*. 15 *BRCA1/2* mutations matched 15 germline *BRCA1/2* mutations identified in blood samples. Two additional *BRCA1/2* mutations, which were not identified in blood samples, were considered as somatic: one *BRCA1* and one *BRCA2* (Table 3). Both somatic mutations *BRCA1* c.1543G > T p.(Glu515*) and *BRCA2* (del ex 22–27) are known pathogenic mutations. Both patients with somatic mutations had a negative family history and did not have a breast cancer.

**Table 3:** *BRCA* mutations in cytological samples. All patients were previously tested for germline mutations from DNA extracted from the peripheral blood samples

Sample No.	% of TC in the sample	Gene	Mutation	AF in CS	Germline vs. somatic mutation ^i,j^	Classification	BIC^b^	HGMD^c^	UMD^d^	LOVD^e^	ClinVar^f^
			HGVS c.DNA^a^ HGVS protein^a^								
1	*70–80%*	*BRCA1*	c.844_850dupT CATTAC ^h^ p.(Gln284Leufs*5)	41%	germline	pathogenic	CI	DM	C	AfF	P
2	*90%*	*BRCA1*	c.116G > A ^h^ p.(Cys39Tyr)	97%	germline	pathogenic	UV	DM	/	AfF	P
3	*70%*	*BRCA1*	deletion of exons 4–9^g^ c.(134 + 1_135–1)_(670 + 1_671–1)del p.?	85%	germline	pathogenic	/	DM	/	/	/
4	*80%*	*BRCA1*	c.1543G > T ^h^ p.(Glu515*)	83%	somatic	pathogenic	/	/	/	/	P
5	*10%*	*BRCA1*	c.3018_3021delTTCA ^h^ p.(His1006Glnfs*17)	48%	germline	pathogenic	CI	DM	C	/	P
6	*30%*	*BRCA1*	c.1687C > T ^h^ p.(Gln563*)	53%	germline	pathogenic	CI	DM	C	AfF	P
7	*90%*	*BRCA1*	c.850C > T ^h^ p.(Gln284*)	94%	germline	pathogenic	/	DM	/	/	P
8	*70–60%*	*BRCA1*	deletion of exons 4–9^g^ c.(134 + 1_135–1)_(670 + 1_671–1)del p.?	80%	germline	pathogenic	/	DM	/	/	/
9	*30–40%*	*BRCA2*	c.4139_4140dupTT ^h^ p.(Lys1381Leufs*8)	55%	germline	pathogenic	/	DM	/	/	P
10	*80%*	*BRCA1*	c.843_846delCTCA ^h^ p.(Ser282Tyrfs*15)	93%	Germline	pathogenic	CI	DM	C	AfF	P
11	*60%*	*BRCA1*	c.843_846delCTCA ^h^ p.(Ser282Tyrfs*15)	84%	germline	pathogenic	CI	DM	C	AfF	P
12	*> 95%*	*BRCA2*	deletion of exons 22–27^g^ c.(8754 + 1_8755–1)_(*1_?)del p.?	80%	somatic	pathogenic	/	DM	/	/	/
13	*50%*	*BRCA1*	c.843_846delCTCA ^h^ p.(Ser282Tyrfs*15)	81%	germline	pathogenic	CI	DM	C	AfF	P
14	*1–3%*	*BRCA1*	c.1687C > T ^h^ p.(Gln563*)	51%	germline	pathogenic	CI	DM	C	AfF	P
15	*< 10%*	*BRCA1*	c.3436_3439delTGTT ^h^ p.(Cys1146Leufs*8)	43%	germline	pathogenic	/	DM	C	/	P
16	*20%*	*BRCA1*	c.844_850dupTCATTAC ^h^ p.(Gln284Leufs*5)	46%	germline	pathogenic	CI	DM	C	AfF	P
17	*40%*	*BRCA1*	c.4065_4068delTCAA ^h^ p.(Asn1355Lysfs*10)	54%	germline	pathogenic	CI	DM	/	UV	P

^a^The description of nucleotide sequence variations is in accordance with HGVS nomenclature. DNA variants are numerated according to NCBI reference sequence NM_007294.3 for *BRCA1* and NM_000059.3 for *BRCA2.* First nucleotide of start codon ATG is numerated as 1

^b^ https://research.nhgri.nih.gov/bic/

^c^ https://portal.biobase-international.com/

^d^http://www.umd.be/BRCA1/ and http://www.umd.be/brca2/

^e^https://lovd.nl/BRCA1 and https://lovd.nl/BRCA2

^f^ https://www.ncbi.nlm.nih.gov/clinvar/

^g^All large deletions were confirmed by MLPA analysis in cytological and tumor sample

^h^All SNV as well as indel were confirmed by Sanger sequencing in blood samples in case of germline mutations, and in cytological and tumor samples in case of somatic mutation

^i^Mutations were defined as germline, when they were detected in patients blood sample with mutation allele frequencies > 45%

^j^Mutations were defined as somatic, when detected in FFPE tumor samples and CF samples and not detected in patients blood sample

TC-tumor cells, AF-allele frequency in the sample, CS-cytological sample, P-pathogenic, CI-clinically important, DM-disease causing mutation, C-casual, AfF-affects function, UV-unknown

#### Histological *BRCA1/2* testing

In 19 patients samples for DNA analysis were obtained at interval cytoreductive surgery (after 4–6 cycles of chemotherapy) in 22 patients during primary surgeries and in 3 during surgical treatment of recurrent disease. Percentage of tumor cells present on FFPE block slide was on average 83.2% (range 65–95). Exactly the same 17 *BRCA1/2* mutations which were identified in cytological samples were identified in histological samples (100% concordance).

No additional somatic mutations were detected in tumors from patients with germline *BRCA* mutations in cytological samples. All patients were previously tested for germline mutations from DNA extracted from the peripheral blood samples (Table 3).

## Discussion

We postulated that *BRCA1/2* mutation analyses from cancer cells derived from cytological samples like ascites, pleural effusion and enlarged lymph nodes would be concordant with *BRCA1/2* mutation analyses from FFPE tumor tissue. There is no doubt that tumor tissue (FFPE) is standard material for initial diagnosis of HGSC. However, in our study the 100% concordance in detection of germline *BRCA*1/2 mutations between all three sample types (CS, FFPE and blood) was confirmed. Moreover, the 100% concordance was also observed between mutation detection in CS and FFPE in two cases of somatic mutations alluding that cytology samples could replace tumor tissue in determining *BRCA1/2* mutation status in patients with HGSC where FFPE is hardly available. This is true when cytological and FFPE tumor samples have expected allele frequency of mutations above frequencies defined as limits of detection of the method used for genotyping (e.g. if tumor cells are heterozygous for mutations, the percentage of tumor cells in the sample should be twice of the limit of detection).

Our data strongly support and upgrade studies from Shah at all, where pleural effusion from 4 HGSC patients was used as cytological sample for DNA extraction and characterization of genomic alterations [18] and Choi et all, where conservation of cancer related genes in ascites cells from 4 ovarian cancer patients was evaluated.

Since ovarian cancers are characterized by complex and changing genetic profile, *BRCA1/2* tumor testing results can potentially vary depending on disease stage and sample site [20]. There is also the possibility that inactivated (mutated) *BRCA1/2* gene can revert to a functional gene during disease treatment and progression, due to somatic reverting mutations (resulting in resistance to therapy to PARP inhibitors). However in our cohort no reverting somatic mutations in *BRCA* genes were detected. As nearly half of patients included in our study did not have recurrence of the disease, this could induce bias, because reverting mutations could not have time to develop yet. Since the number of patients included in the study was too low to optimally address this issue, the reverting mutation analysis was not the aim of this study. Continuation of this study is already taking place to eliminate this bias.

Together with before mentioned data and possibility for mutation reversal during cancer progression and treatments, ascites cells are proven to be a very good resource for identifying genomic alterations [17, 30]. When using cytologicaly derived cancer cells DNA to detect *BRCA1/2* mutation, both germline and somatic *BRCA1/2* mutations are identified. Results are independent on tumor sampling site. This is an important advantage, as potentially only one test needs to be performed to identify all patients with deleterious *BRCA1/2* variants that may benefit from new molecular targeted treatment. To distinguish between germline and somatic *BRCA* mutations, additional blood test must be performed.

Incidence of *BRCA* mutations in our study was 34%, which is among the highest reported. An estimated 16–39.2% of OC patients are likely to have either germline or somatic *BRCA1/2* mutation according to the literature [6]. The highest reported incidence was from the study of Petrillo et all, were incidence of *BRCA* mutations in HGSC patients was 39.2%. Unfortunately, *BRCA* mutations in their study were not specified to germline or somatic, so exact comparison with our study is not possible [10]. Our results confirmed results of Cvelbar et all who analyzed the incidence of germline *BRCA* mutations in ovarian cancer patients younger than 50 years in Slovenia and found 33.3% incidence of *BRCA* mutations [31]. Our study presents first data about the incidence of somatic mutations among HGSC patients in Slovenia. The low incidence of 4.5% of somatic mutations is in agreement with the results of other international studies. Study of Integrated Genomic Analyses of Ovarian Carcinoma reported 3% incidence of somatic mutations and Geisler et all reported 6,8% incidence of somatic mutations, though in their study where not only HGSCs, but all epithelial ovarian cancers assessed for *BRCA* germline and somatic mutations [8, 32].

Since 34% of our patients had a germline *BRCA1/2* mutation, it is surprising that only 8/44 of them had a positive family history of breast and ovarian cancer. However, this is somehow in agreement with the results of Alsop et all who also reported that 44% of *BRCA* positive patients in Australia presented with no family history of breast and ovarian cancer [7].

Beside a positive family history, multiple primary cancers is another important aspect of hereditary cancer syndromes. In our study we had a complete data for 32 patients and 6 (18,7%) of them had an additional breast cancer diagnosed before HGSC was diagnosed. Family history was positive in 4/6 (66,7%) of them. Mean age (at the time of diagnosis of OC?) of patients with germline *BRCA1/2* was 56,9 years (range 43–68). As expected, mean age at the time of diagnosis of *BRCA* negative patients was higher, 61,7 years (range 40–77).

According to the NCCN guidelines genetic testing should be considered in appropriate high risk individuals where it will impact the medical management of the tested individual and/or their family members at risk. Ideally the probability of mutation detection should be more than 10% according to the family tree [33]. Since germline mutation rate according to the results of our study and also results of other already mentioned published studies by far exceed 10%, we are facing a similar situation like in patients with medullary thyroid cancer, where genetic counselling and testing is recommended in all patients and not only in those with a positive family history [34]. All patients with epithelial ovarian cancer (including HGSC) should be offered genetic counselling and testing regardless of family history.

Results of our study demonstrated a 100% concordance of *BRCA1/2* testing from peripheral venous blood and different cytological samples for detecting a germline mutation in HGSC patients. As such, this approach could simplify and reduce testing rates in OC patients. Patients with negative cytological or histological *BRCA* test results do not need additional genetic testing for germline BRCA mutations. Only patients who have a positive cytological or histological *BRCA* test result require additional genetic testing to rule out or confirm germline mutation. Considering before mentioned *BRCA* mutations frequency in HGSC cancer patients, more than 70% of HGSC patients would need only cytologicaly derived *BRCA* mutation test. For 100 cytological or histological samples an additional 30 tests (130 tests altogether) have to be done to differentiate between somatic and germline mutations. On the other hand, if we start with 100 germline mutation tests first and 30 of them will result positive, we need additional 70 tests (170 tests altogether) to differentiate between somatic and germline mutations. Not to mention that by such an approach all patients need to be genetically counselled. Histological tumor sample (usually provided from initial surgery) is therefore only needed for diagnosis of HGSC, whereas cytologic tumor sample offers a good alternative for *BRCA* testing.

In this regard, evidences presented in this study strongly suggest clinical significance of cytological samples as a valuable tool for widely use in genetic *BRCA* testing. Simple, rapid and cost-effective methods (at least 70% reduction in genetic counselling and genetic testing) of molecular testing, which take full advantage of the limited materials available in the clinical setting, such as cytology samples, are urgently required. This is particularly important in cases of frequent recurrences of disease with limited options for surgical treatment and limited availability of histopathologic samples. Cytological material is already routinely used in clinical setting to confirm recurrence/progression of the disease. Regarding to our study results, it could be used for *BRCA* re-testing after each recurrence/progression of the disease. Since HGSC patients most of the time have multiple recurrences of disease and become chemoresistant, subjecting them to molecular profiling as part of their care during disease progression, enables clinicians to select targeted personalized therapies according to the mutations detected in their tumors before every treatment enrolment. With maximum confidence data, this will hopefully become a part of clinical pathways in the future.

## Conclusions

There is a 100% concordance between *BRCA1/2* mutation testing from cytological and histological samples. Moreover, in all patients with a germline *BRCA1/2* mutation there is also a 100% concordance between *BRCA1/2* mutation testing from cytological and blood samples.

Cytological samples with adequate percentage of tumor cells are equivalent to FFPE samples in determining *BRCA* mutations and could replace FFPE in *BRCA 1/2* gene mutation profiling in HGSC patients. Additional benefits when cytological samples are tested, could be in faster processing of genotyping and earlier informative report for clinician as well as in potentially reduced number of genetic testing.

## Abbreviations

AF: Allele frequency in the sample
AfF: Affects function
AS: Ascites
C: Casual
CI: Clinically important
CS: Cytological samples
DM: Disease causing mutation
ECC: Endogenous creatinine clearance,
ELN: Enlarged lymph nodes
FFPE: Formalin-fixed paraffin-embedded
HE: Hematoxylin and Eosin,
HG: Homologous recombination
HGSC: High grade serous cancer
Indel: Insertion and deletions
ITH: Intratumoral genetic heterogeneity
NGS: Next Generation Sequencing
OC: Ovarian cancer
P: Pathogenic
PARPi: Poly(ADP-ribose) polymerase inhibitors
PE: Pleural effusion
PFS: Progression-free survival
SNV: Single nucleotide variants
TC: Tumor cells
TSCA: TruSeq Custom Amplicon Kit
TSCancer: TrueSight Cancer panel
UV: Unknown
